# N6-methyladenosine modification of the subgroup J avian leukosis viral RNAs attenuates host innate immunity via MDA5 signaling

**DOI:** 10.1371/journal.ppat.1013064

**Published:** 2025-04-08

**Authors:** Mengmeng Yu, Li Zhang, Ying Wang, Suyan Wang, Yongzhen Liu, Peng Liu, Yuntong Chen, Ru Guo, Lingzhai Meng, Tao Zhang, Wenrui Fan, Xiaole Qi, Yulu Duan, Yanping Zhang, Hongyu Cui, Yulong Gao

**Affiliations:** 1 Avian Immunosuppressive Diseases Division, State Key Laboratory for Animal Disease Control and Prevention, Harbin Veterinary Research Institute, The Chinese Academy of Agricultural Sciences, Harbin, PR China; 2 Jiangsu Co-innovation Center for Prevention and Control of Important Animal Infectious Disease and Zoonoses, Yangzhou University, Yangzhou, PR China; 3 National Poultry Laboratory Animal Resource Center, Harbin, PR China; National Institutes of Health-NIAID, UNITED STATES OF AMERICA

## Abstract

Subgroup J avian leukosis virus (ALV-J), a retrovirus, elicits immunosuppression and persistent infections in chickens. Although it is widely acknowledged that ALV-J can evade the host’s innate immune defenses, the mechanisms behind this immune evasion remain elusive. N6-methyladenosine (m^6^A), the most prevalent internal RNA modification, plays a role in innate immune evasion. Our research identified ALV-J as an inefficient stimulator of innate immunity *in vitro* and *in vivo*, with its genomic RNA featuring m^6^A modifications predominantly in the envelope protein (*Env)* region and 3′ untranslated region (*3*′*UTR*). To elucidate the functional consequences of m^6^A modification, we subsequently generated m^6^A-deficient ALV-J through its culturing in the DF-1 overexpressing fat mass and obesity-associated protein (FTO) cells. The m^6^A-deficient ALV-J virus, or its RNAs significantly enhanced *IFN-*β production compared to the wild-type (wt) ALV-J, suggesting a pivotal regulatory function of m^6^A modifications in modulating innate immune response. Mechanistically, the m^6^A modification of the ALV-J genomic RNA directly impacted its recognition by MDA5, weakening its binding and ubiquitination and attenuating IFN-β activation. Moreover, m^6^A-deficient ALV-J, created by inducing mutations in m^6^A sites within *Env* and *3*′*UTR*, exhibited reduced replication capacity and elevated IFN-β expression in host cells. Importantly, this phenomenon was abolished in MDA5-knockout DF-1 cells, further demonstrating the core role of MDA5. These data demonstrate that m^6^A modification of ALV-J genomic RNA dampens the host’s innate immune response through MDA5 signaling pathway.

## Author summary

ALV-J has a long latency and can cause persistent infections with continuous viral shedding, leading to severe immunosuppression and tumor induction in chickens and consequently significant economic losses to the poultry industry. However, the mechanisms by which ALV-J escapes the host’s innate immune recognition and clearance and achieves persistent infection remain elusive. In this study, we systematically identified, for the first time, that the m^6^A modifications of ALV-J genomic RNA were primarily concentrated in the *Env* and *3*′*UTR* regions and that this modification is crucial in the viral evasion of the host’s innate immunity. Furthermore, we parsed the in-depth mechanism that the m^6^A modifications of ALV-J RNA mediate the evasion of RNA recognition by the host’s innate immunity through the MDA5 signaling pathway to attenuate IFN-β expression. Our findings reveal the significance of m^6^A modifications in the immune evasion strategies of ALV-J and offer a novel perspective on the complex interactions between the virus and the host’s innate immune response.

## Introduction

Avian leukosis (AL) is a general term for contagious tumor diseases caused by the avian leukosis virus (ALV) [1]. The ALV has been classified into 11 subgroups (A–K) based on virus envelope genes and serological cross-neutralization experiment [2–4]. Among them, ALV-J has the strongest transmission ability and pathogenicity and induces the formation of tumors such as myelocytoma and hemangioma [3,5]. ALV-J, as a retrovirus, is primarily transmitted vertically. Young chicks are the most susceptible to ALV-J, however, most infected flocks do not have tumors until around the egg production stage. After infection, ALV-J has a long latency period, and there is persistent infection and uninterrupted shedding of the virus, causing critical damage to the production performance of infected chicks and threatening the healthy development and seed security of the poultry industry [5–7].

When the virus infects the host, it activates the host’s innate immunity, which is the first line of defense for the host against viral infections. The innate immune response primarily involves the host’s recognition of pathogens through various pattern recognition receptors (PRRs) [8] and the induction of the host to produce type I interferon or other antiviral factors, protecting the host [9–11]. To better survive and replicate in the host, the virus has evolved various strategies to evade the innate immune response of the host, such as targeting negative regulatory factors of interferon signaling pathways using viral structural or non-structural proteins, expressing viral miRNAs or regulating host miRNA expression to target host proteins and suppress host antiviral responses, or the special structure and epitranscriptomic modification of viral nucleic acids and proteins to PRRs [12–14]. Among them, the modification of viral nucleic acids has received significant attention recently [15].

RNA has over 160 modification types, among which N6-methyladenosine (m^6^A) modification is the most common mRNA epitranscriptomic modification, with over 25% of mammalian transcripts exhibiting m^6^A modification [16–18]. m^6^A modification is reversible and is primarily regulated by two classes of intracellular proteins. The “writers” protein of m^6^A methylation catalyzes the methylation of m^6^A on specific motifs RRACH and primarily comprises two subunits, namely METTL3 (catalytic enzyme) and METTL14 (an allosteric activator) [19–21]. The “erasers” of m^6^A modification include RNA demethylase FTO and ALKBH5 [22–24], which can selectively remove m^6^A modification. The m^6^A modification is primarily recognized by m^6^A “reader” proteins (YTHDF1, YTHDF2, YTHDF3, and YTHDC1) [25,26]. The binding of m^6^A “reader” proteins to m^6^A modification markedly influences RNA transport, stability, localization, and translation [26–28]. Viruses are strictly intracellular parasites that can acquire m^6^A modification within the host. Since the 1970s, m^6^A modifications have been found in the genomes of influenza A virus, simian virus 40, human immunodeficiency virus (HIV), and adenovirus with nuclear replication [29–31]. m^6^A modification can mask their nucleic acids to evade the monitoring by the host’s innate immunity and achieve persistent infection [32–34].

Similarly, ALV-J is an intracellular nucleus-replicating avian virus that evades the host’s immune surveillance, establishes persistent infection in the host, and induces tumor formation [12,35]. Recent reports indicated that the RNA m^6^A modification is essential in regulating the immune system. However, data confirming the m^6^A modification of ALV-J genomic RNA and explaining its mechanism in the innate immune response to ALV-J are lacking. In this study, we found that the envelope protein (*Env*) and 3′untranslated region (*3*′*UTR*) regions of the ALV-J genome are enriched with m^6^A modifications, which can reduce PRRs sensing and IFN-β production. We also observed that the m^6^A modification of viral RNA primarily inhibits IFN-β expression by weakening MDA5 recognition of viral RNA, diminishing the binding between MDA5 and viral RNA and reducing MDA5 ubiquitination. In addition, m^6^A-deficient ALV mutants induced a stronger IFN response *in vivo* and *in vitro*, attenuating the replication ability.

## Results

### ALV-J is an inefficient stimulator of innate immunity

**Fig 1:**
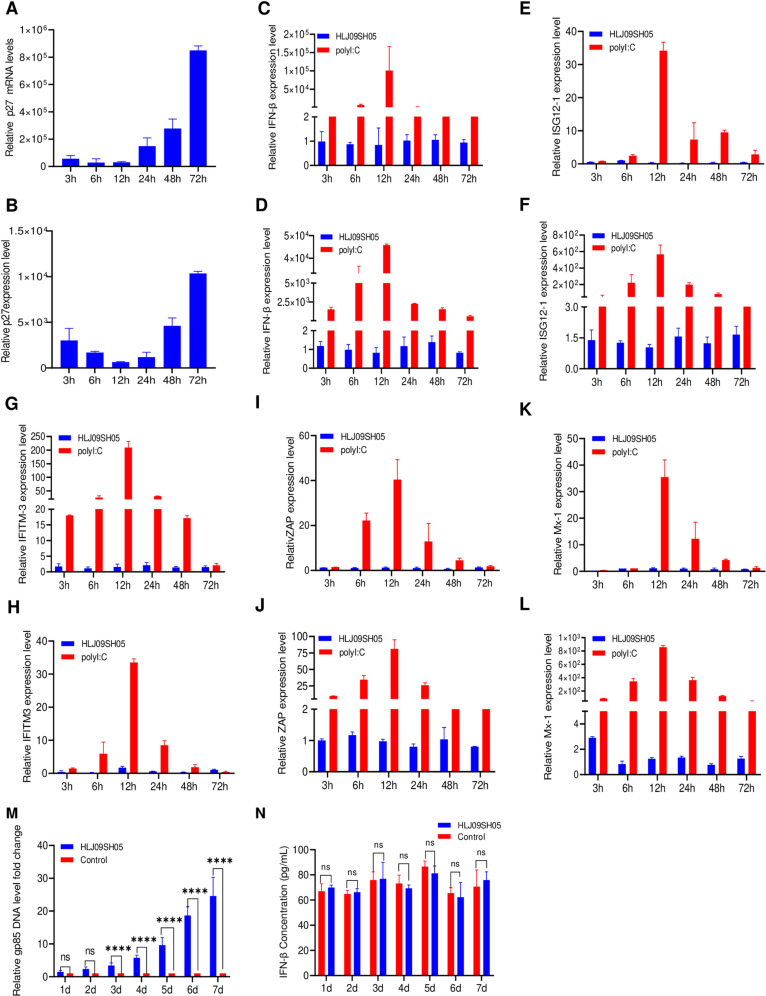
ALV-J does not effectively activate the innate immune response during viral replication. (A-L) Following infection with the prevalent virus strain HLJ09SH05, DF-1 cells and primary macrophages were assessed the mRNA expression levels of p27 in DF-1 cells (A) and chicken primary macrophages (B), *IFN-β* in DF-1 cells (C) and chicken primary macrophages (D), *ISG12-1* in DF-1 cells (E) and chicken primary macrophages (F), *chIFITM3* in DF-1 cells (G) and chicken primary macrophages (H), *ZAP* in DF-1 cells (I) and chicken primary macrophages (J), and *Mx-1* in DF-1 cells (K) and chicken primary macrophages (L) using the RT-qPCR at 3 to 72 hpi. (M and N) Similarly, 1-day-old SPF chickens were intraperitoneally inoculated with 10^4^ TCID_50_ HLJ09SH05 strain, and viral loads in whole-blood samples were measured using RT-qPCR (M), and the *IFN-β* was also evaluated using chicken IFN-β ELISA kit. The relative amounts of *p27*, *IFN-β, ISG12-1*, *IFITM3*, *ZAP*, and *Mx-1* mRNA were normalized to the *GAPDH* mRNA levels in each sample, and the fold differences were compared with those in the mock samples. *:p < 0.05, **: p < 0.01, ***: p < 0.001, ****: p < 0.0001; ns: no significant difference.

To understand the innate immune response triggered by ALV-J *in vivo* and *in vitro*, we initially inoculated ALV-J strain HLJ09SH05 into DF-1 cell lines and chicken primary macrophages. Real-time quantitative polymerase chain reaction (RT-qPCR) results showed that HLJ09SH05 successfully infected and was replicated in both cell types (Fig 1A and 1B). However, it did not stimulate Interferon-β (*IFN-β*) (Fig 1C and 1D), IFN-α-stimulated gene 1 (*ISG12–1*) (Fig 1E and 1F), IFN-induced transmembrane protein 3 (*IFITM3*) (Fig 1G and 1H), Zinc-finger antiviral protein (*ZAP*) (Fig 1I and 1J), or single-copy antiviral gene (*Mx-1)* (Fig 1K and 1L) production. This was in contrast to the robust expression induced by transfection with poly (I:C) at 1ug/ml in both cell types. Furthermore, we assessed the IFN-β expression of chickens infected with the ALV-J strain HLJ09SH05. The serum from 1 to 7 days post-inoculation (dpi) was collected to be detected virus viremia and IFN-β induction. These results indicated that HLJ09SH05 successfully replicated in the chicken, with viremia detectable on 3 dpi (Fig 1M), however, HLJ09SH05 induced no significant increase in IFN-β production compared with that in the control group (Fig 1N). Taken together, these results suggest that ALV-J, while capable of replication, does not effectively trigger a robust innate immune response *in vivo* and *in vitro,* suggesting that ALV-J is a poor inducer of innate immune responses.

### ALV-J genomic RNA is modified by m^6^A methylation

**Fig 2:**
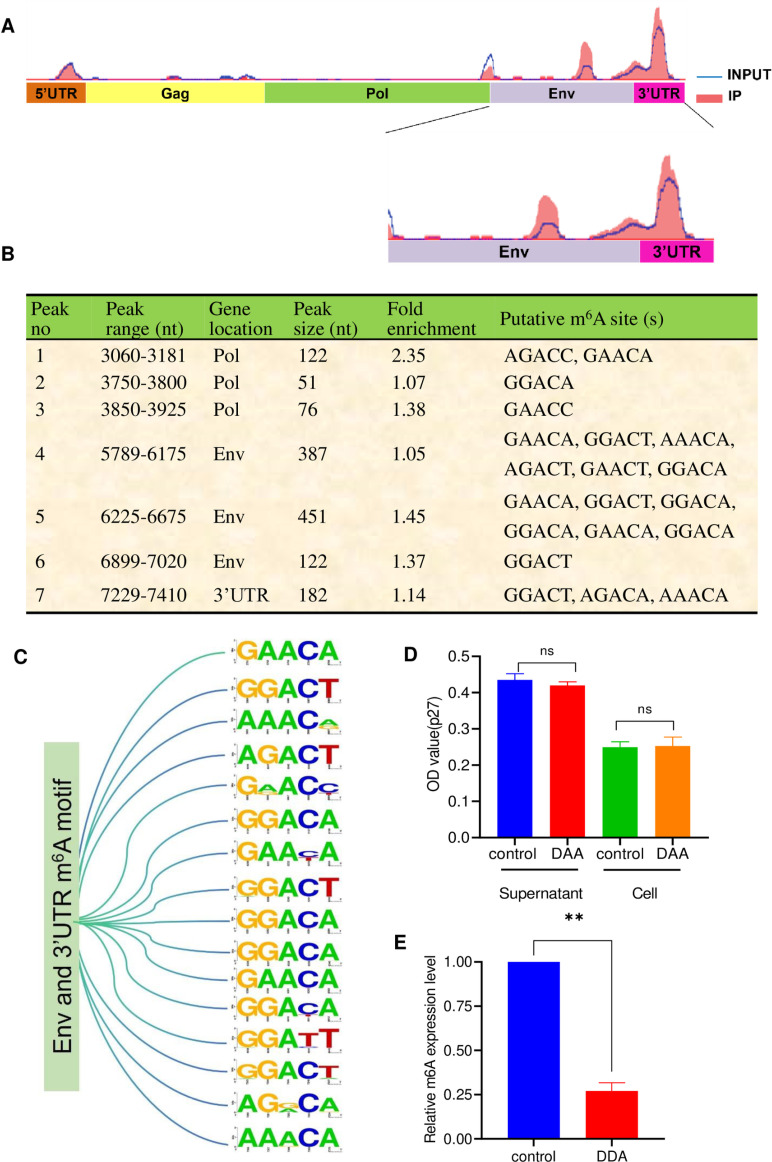
ALV-J genome RNAs are m^6^A-methylated. (A and B) Distribution of m^6^A peaks in the ALV-J genome. The total RNA of DF-1 cells infected with HLJ09SH05 was extracted at 4 dpi and subjected to m^6^A-specific antibody immunoprecipitation, followed by high-throughput sequencing (MeRIP-seq). Burgundy red areas illustrate the distribution of m^6^A immunoprecipitation reads aligned to the ALV-J mRNAs, while the baseline signal from input samples is depicted as a continuous line. (B) The distribution area of m^6^A modification sites in the genomic RNA of HLJ09SH05 was demonstrated, and the presence of m^6^A motifs (RRACH) was searched for in the identified m^6^A peaks, verifying the predicted m^6^A sites. (C) Conservation analysis of 16 m^6^A motifs within the *Env* and *3’UTR* regions. The Sequence Logos of 16 m^6^A motifs within the *Env* and *3’UTR* regions were drawn by Web Logo software (http://weblogo.berkeley.edu/logo.cgi).All sequences of ALV-J strains were obtained from GenBank. (D) DAA treatment did not affect the replication and release of the virus. DF-1 cells were treated with DMSO as control or DAA (10 μM) for 4 h and transfected with the HLJ09SH05. The cell supernatants were collected at 4 dpi. (E) DAA treatment reduced the m^6^A modifications of ALV-J RNA. DF-1 cells were treated with DMSO as control or DAA (10 μM) for 4 h and transfected with the HLJ09SH05. ALV-J in the supernatants was collected at 4 dpi.

To systematically determine whether the ALV-J genome exhibits m^6^A modifications and their possible locations in the genome, the mRNAs from HLJ09SH05-infected DF-1 cells were subjected to m^6^A immunoprecipitation, followed by high-throughput sequencing (MeRIP-seq). Sequencing results indicated that there were seven m^6^A peaks in the HLJ09SH05 genome, most of which were at the *Env* and *3*′*UTR* regions (Fig 2A and 2B). Sequence analysis revealed that 87.5% (14/16) of the predicted m^6^A motifs within the *Env and 3’UTR regions* are relatively conserved (Fig 2C). To further confirm the presence of m^6^A modification in the ALV-J genome, we also investigated the pharmacological inhibition of m^6^A modification of ALV-J RNA using 3-deoxyadenosine (DAA), a known inhibitor of S-adenosylhomocysteine (SAH) hydrolase that catalyzes the reversible hydrolysis of SAH to adenosine and homocysteine. DAA causes the accumulation of SAH, which in turn increases the ratio of SAH-to-S-adenosyl methyl thionine (SAM), which is a substrate for m^6^A modification, and subsequently inhibits SAM-dependent methyltransferases. The DF-1 cells were treated with 10 μM of the m^6^A-modified inhibitor DAA and inoculated with HLJ09SH05. Compared with untreated DF-1 cells, DAA treatment of DF-1 cells did not affect the replication and release of HLJ09SH05 (Fig 2D), however, it reduced m^6^A modification levels in HLJ09SH05 RNA by approximately 73% (Fig 2E). These results indicate that the ALV-J genome exhibits the m^6^A modification, which is primarily enriched in the *Env* and *3*′*UTR* regions.

### m^6^A-deficient ALV-J induces high IFN-β expression

**Fig 3:**
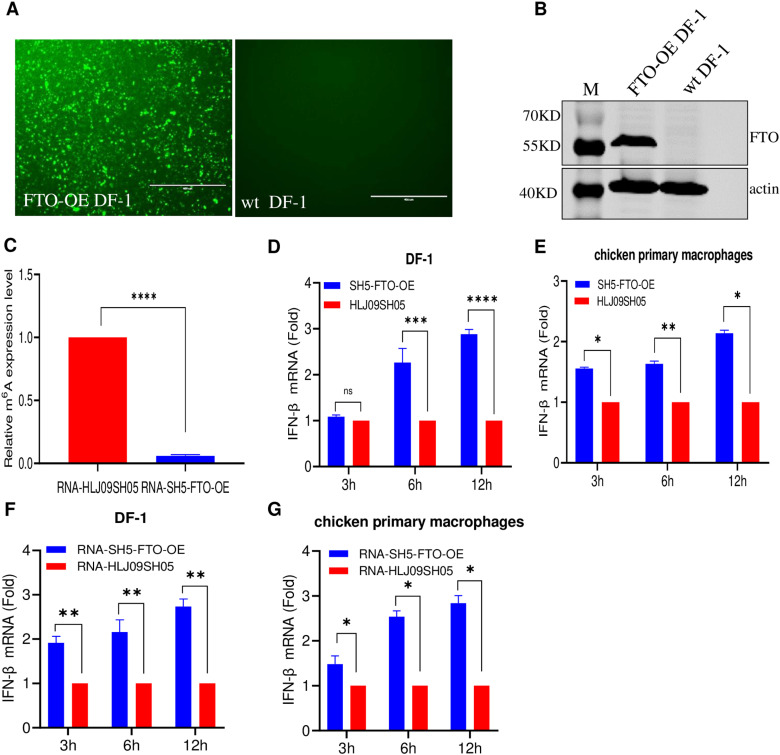
m^6^A-deficient ALV-J and its virion RNAs induce higher *IFN-β* expression. (A and B) Construction of FTO-OE DF-1 cell line. (A) IFA. The DF-1 cells and FTO-OE DF-1 cells were detected with the Flag monoclonal antibody and analyzed using a fluorescence microscope; the DF-1 cells were the reference. Scale bar: 400 μm. (B) Western blotting. The lysates of the DF-1 cells and FTO-overexpressing DF-1 cells were detected with the Flag monoclonal antibody. (C) Relative m^6^A levels in the virion RNA from ALV grown on FTO-OE DF-1 andwt DF-1 cells. We inoculated ALV-J into FTO-OE DF-1 cell lines to obtain viruses with m^6^A deficiency. The m^6^A levels for each virion RNA were quantified using an m^6^A methylation kit. (D) m^6^A-deficient and wt ALV-J induced *IFN-β* mRNA expression after infecting DF-1 cells. DF-1 cells were infected with m^6^A-deficient or wt ALV-J. At 3, 6, and 12 hpi, cells were collected for the analysis of *IFN-β* mRNA levels using RT-qPCR. (E) m^6^A-deficient and wt ALV-J induced *IFN-β* mRNA expression after infecting chicken primary macrophage cells. Chicken primary macrophage cells were infected with m^6^A-deficient or wt ALV-J. At 3, 6, and 12 hpi, cells were collected for the analysis of *IFN-β* mRNA levels using RT-qPCR. (F) The RNAs of m^6^A-deficient and wt ALV-J induced *IFN-β* mRNA expression after transfecting in DF-1 cells. DF-1 cells were transfected with 10^8^ copies of the m^6^A-deficient or wt ALV-J RNA. Cells were collected at 3, 6, and 12 hpt to analyze *IFN-β* mRNA levels using RT-qPCR. (G) The RNAs of m^6^A-deficient and wt ALV-J induced *IFN-β* mRNA expression after transfecting in chicken primary macrophage cells. chicken primary macrophage cells were transfected with 10^8^ copies of the m^6^A-deficient or wt ALV-J RNA. Cells were collected at 3, 6, and 12 hpt to analyze *IFN-β* mRNA levels using RT-qPCR.

To determine the role of viral m^6^A methylation in ALV-J-induced innate immunity, we first constructed a FTO-overexpressing DF-1 cell line (FTO-OE DF-1), which is an eraser protein that can remove m^6^A modification on mRNAs. The indirect immunofluorescent assay (IFA) results indicated that FTO-OE DF-1 cells expressed FTO and showed specific green fluorescence. However, the wt DF-1 cells displayed no fluorescence (Fig 3A). Similarly, western blotting results showed that FTO-OE DF-1 cells produced approximately 56-kDa band with the anti-FLAG monoclonal antibody (Fig 3B). These results showed that the FTO-OE DF-1 cell line was successfully constructed. To obtain an m^6^A-deficient virus, the FTO-OE DF-1 cells were infected with HLJ09SH05, and the cell supernatant was collected at 7 dpi. The Enzyme-linked immunosorbent assay (ELISA) of the m^6^A levels showed that the RNA of the virion harvested from FTO-OE DF-1 cells was demethylated, with approximately 90% decrease in the m^6^A modification levels compared with that of HLJ09SH05 RNA (Fig 3C). The m^6^A-deficient virus was named SH5-FTO-OE.

To determine the effect of m^6^A modification of ALV RNA on *IFN-β* induction during viral infection, SH5-FTO-OE and HLJ09SH05 were separately incubated with DF-1 cells. RT-qPCR results showed that SH5-FTO-OE induced approximately 2.3- and 2.85-fold higher *IFN-β* expression levels compared to that of HLJ09SH05 at 6 and 12 h post-inoculation (hpi) (Fig 3D). To validate the observations in the DF-1 cells, we also assessed *IFN-β* expression levels in chicken primary macrophages induced by SH5-FTO-OE and HLJ09SH05. The results also showed that SH5-FTO-OE induced 1.5–2.1-fold higher *IFN-β* mRNA levels compared to that of HLJ09SH05 at 3–12 hpi (Fig 3E). To eliminate the effects of ALV-J replication and viral protein synthesis on the induced *IFN-β* expression levels, we separately transfected the RNAs of SH5-FTO-OE and HLJ09SH05 into DF-1 cell and found that the viral RNA of SH5-FTO-OE also induced a significantly 1.9–2.7-fold higher *IFN-β* expression levels compared to that of the HLJ09SH05 RNA at 3–12 hpi (Fig 3F). Similar results were also observed in chicken primary macrophages (Fig 3G). Taken together, these results indicate that m^6^A-deficient ALV-J SH5-FTO-OE can induce significantly higher levels of *IFN-β* compared to that of m^6^A-sufficient HLJ09SH05.

### m^6^A modification of ALV-J RNA regulates the recognition by MDA5

**Fig 4:**
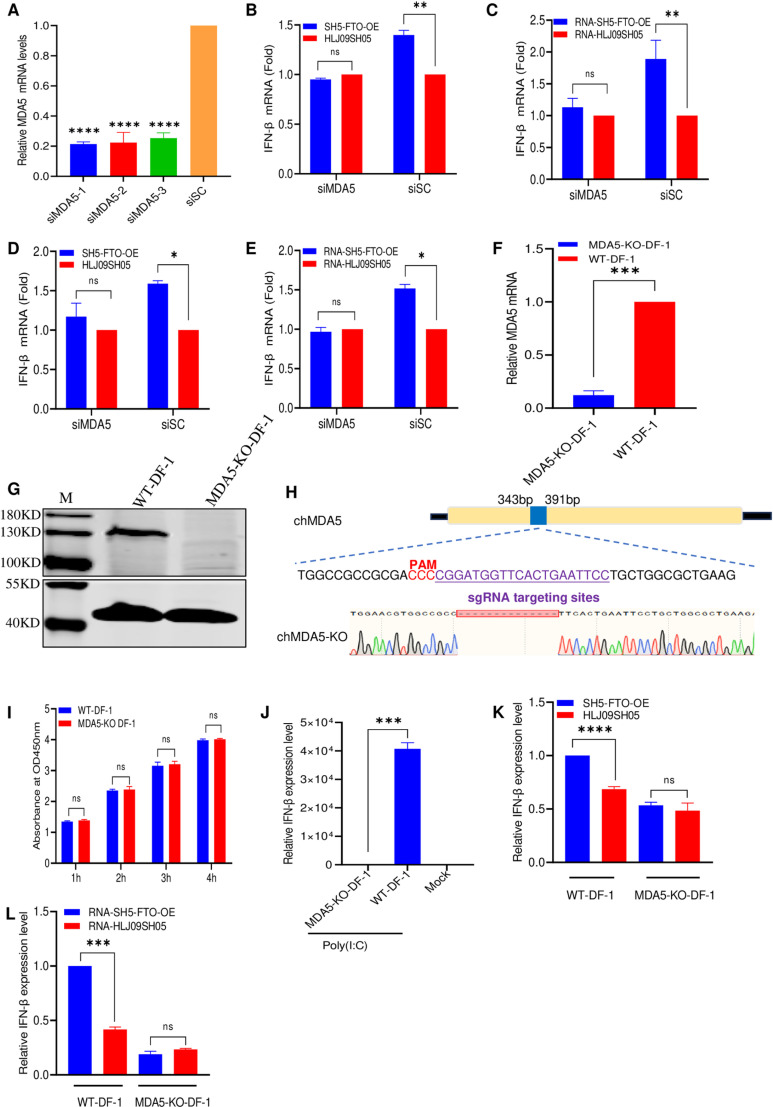
MDA5 is the primary RNA sensor that recognizes m^6^A-deficient ALV-J RNAs. (A) Validation of the optimal siRNA targeting MDA5 using RT-qPCR. (B) The effect of MDA5 knockdown on m^6^A-deficient virus-induced *IFN-β* mRNA expression in DF-1 cells. The DF-1 cells were transfected with 2 μg siMDA5-1 or negative siRNA control siSc. for 24 h and were subsequently infected with m^6^A-deficient or wt ALV-J for 24 h. (C) The effect of MDA5 knockdown on the RNA of m^6^A -deficient virus-induced *IFN-β* mRNA levels in DF-1 cells. The DF-1 cells were transfected with 2 μg siMDA5-1 or negative siRNA control siSc. for 24 h and were subsequently transfected with 10^8^ copies of m^6^A-deficient or wt ALV-J for 12 h. (D) The effect of MDA5 knockdown on m^6^A-deficient virus-induced *IFN-β* mRNA expression in chicken primary macrophages. The chicken primary macrophages were transfected with 2 μg siMDA5-1 or negative siRNA control siSc. for 24 h and were subsequently infected with m^6^A-deficient or wt ALV-J for 24 h. (E) The effect of MDA5 knockdown on the RNA of m^6^A -deficient virus-induced *IFN-β* mRNA levels in chicken primary macrophages. The chicken primary macrophages were transfected with 2 μg siMDA5-1 or negative siRNA control siSc. for 24 h and were subsequently transfected with 10^8^ copies of m^6^A-deficient or wt ALV-J for 12 h. (F) The MDA5 mRNA level of DF-1 and MDA5-KO DF-1 cell lines. (G) Western Blotting. The DF-1 and MDA5-KO DF-1 cell lines were detected by MDA5 antibody. (H) Sequence analysis of DF-1 and MDA5-KO DF-1 cell lines. (I) Cell viability of DF-1 and MDA5-KO DF-1 cell lines. (J) Poly (I:C) stimulated *IFN-β* production in MDA5-KO and DF-1 cells. Similarly, 1 μg/mL of poly (I:C) was transfected into MDA5-KO DF-1 and DF-1 cells, and after 12 h, the mRNA levels of *IFN-β* were evaluated using RT-qPCR. (K) m^6^A-deficient and wt ALV-J induced *IFN-β* mRNA expression after infecting cells. DF-1 and MDA5-KO DF-1 cell lines were infected with m^6^A-deficient or wt ALV-J. At 12 hpi, cells were collected to analyze *IFN-β* mRNA levels using RT-qPCR. (L) The RNAs of m^6^A-deficient and wt ALV-J induced *IFN-β* mRNA expression after transfecting in cells. DF-1 and MDA5-KO DF-1 cell lines were transfected with 10^8^ copies of the m^6^A-deficient or wt ALV-J RNA. At 12 hpt, cells were collected to analyze *IFN-β* mRNA levels using RT-qPCR.

Previous research has demonstrated that MDA5 and TLR7 recognize the viral RNA of ALV when ALV infects host cells [18]. To investigate how ALV-J evades innate immune recognition through genomic m^6^A modification, we first selected three siRNAs for silenced TLR7. RT-qPCR results indicated that siTLR7–1 significantly reduced TLR7 expression levels (S1A Fig). Subsequently, SH5-FTO-OE and HLJ09SH05 were separately inoculated with siTLR7–1-transfected or siSC-transfected DF-1, and the *IFN-β* levels were determined using RT-qPCR. The results showed that SH5-FTO-OE and its RNA induced higher levels of IFN-β expression in siTLR7 DF-1 and wt DF-1 cells, compared to HLJ09SH05 (S1B Fig) and its RNA (S1C Fig). These results indicate that TLR7 does not play a role in recognizing the m^6^A-deficient viral genome of ALV-J. Next, we also investigated the potential role of MDA5 in sensing m^6^A-deficient ALV RNA. we first selected three siRNAs for silenced MDA5. RT-qPCR results indicated that three siMDA5s significantly reduced MDA5 expression levels (Fig 4A). Subsequently, SH5-FTO-OE and HLJ09SH05 were separately inoculated with siMDA5–1-transfected or siSC-transfected DF-1 cells, and the *IFN-β* levels were determined using RT-qPCR. The results indicated that SH5-FTO-OE triggered higher *IFN-β* levels compared to that of HLJ09SH05 in siSC-transfected DF-1 cells, as expected (Fig 4B). However, SH5-FTO-OE and HLJ09SH05 induced similar *IFN-β* expression levels in siMDA5–1-transfected DF-1 cells (Fig 4B). To further validate this result, the RNAs of both viruses were separately transfected into siMDA5–1-transfected or siSC-transfected cells, and the *IFN-β* expression levels were determined. Consistent with the results of virus infection, when MDA5 was knocked down, the RNA of m^6^A-deficient viruses could not induce high *IFN-β* expression levels (Fig 4C). Similarly, we inoculated siMDA5–1-transfected and siSC-transfected primary macrophages with HLJ09SH05 and SH5-FTO-OE or transfected them with the RNAs of HLJ09SH05 and SH5-FTO-OE to determine *IFN-β* expression levels using RT-qPCR. The results showed that there were no differences in the *IFN-β* expression levels induced by SH5-FTO-OE and its RNA compared to those induced by HLJ09SH05 and its RNA in siMDA5–1-transfected chicken primary macrophages (Figs 4D and 4E). In contrast, in siSC- transfected chicken primary macrophages, SH5-FTO-OE and its RNA induced *IFN-β* expression, which were 1.59- (Fig 4D) and 1.51-fold (Fig 4E) of those induced by HLJ09SH05 and its RNA, respectively. These results suggest that MDA5 is the cellular sensor for detecting m^6^A-deficient ALV-J RNA.

To further confirm the results of the MDA5 knockdown, we constructed the MDA5-knockout (KO) DF-1 cell line with CRISPR/Cas9. RT-qPCR result showed that MDA5 was barely detectable in the MDA5-KO DF-1 cell line (Fig 4F). Western blotting results demonstrated that MDA5 was undetectable in MDA5-KO DF-1 cells when using the MDA5 monoclonal antibody prepared by Sino Biological, whereas MDA5 could be detected in DF-1 cells (Fig 4G). The sequence results showed that the 14 nucleotides at the N-terminus of the MDA5 open reading frame from the MDA5-KO DF-1 cell line were deleted (Fig 4H). The cell counting kit-8 (CCK8) assay also indicated that MDA5-KO did not affect cell viability (Fig 4I). Additionally, we transfected poly (I:C) into MDA5-KO and wt DF-1 cells and evaluated the *IFN-β* levels. These results showed that MDA5-KO DF-1 cells did not induce robust *IFN-β* expression in response to poly (I:C) stimulation (Fig 4J). These results demonstrated the successful construction of the MDA5-KO DF-1 cell line. Subsequently, SH5-FTO-OE and HLJ09SH05 were inoculated into MDA5-KO and wt DF-1 cells. Similar to HLJ09SH05, SH5-FTO-OE did not induce high *IFN-β* expression levels in MDA5-KO DF-1 cells (Fig 4K). However, it triggered a 1.5-fold increase in *IFN-β* expression levels in wt DF-1 cells. To further validate this result, the RNAs of both viruses were separately transfected into MDA5-KO and wt DF-1 cells. Consistent with the results of virus infection, the *IFN-β* expression levels of the RNAs from the two viruses in MDA5-KO DF-1 cells were no discernible difference (Fig 4L). However, the *IFN-β* expression induced by SH5-FTO-OE RNA in wt DF-1 cells was 2.4 times higher than those induced by HLJ09SH05 RNA (Fig 4L). These results suggest that MDA5 is a pivotal cellular sensor for detecting m^6^A-deficient ALV-J RNA.

### m^6^A-deficient viral RNA enhances RNA recognition by and ubiquitination of MDA5

**Fig 5:**
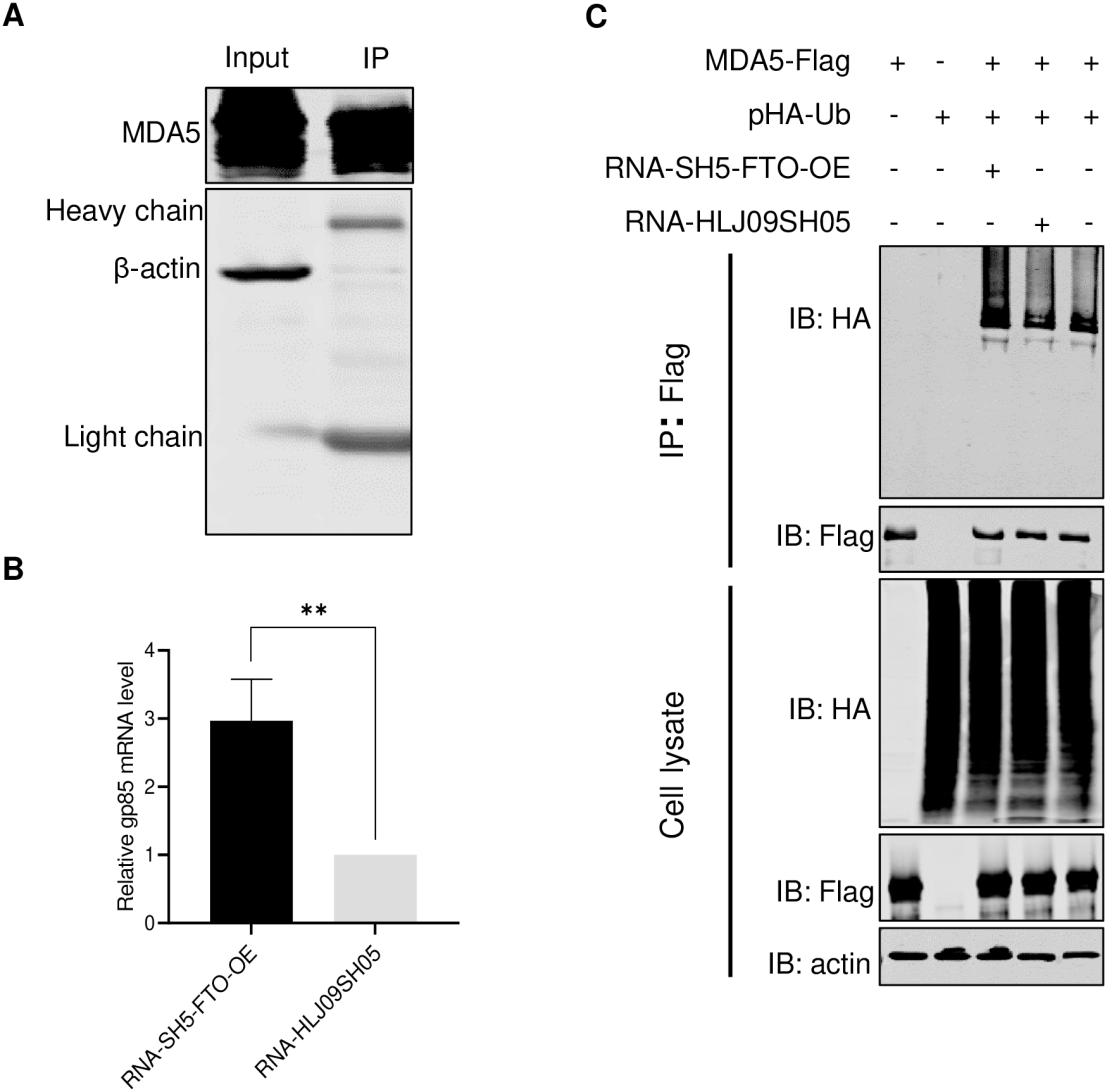
RNA of the m^6^A-deficient virus enhances the binding capacity of MDA5 and its ubiquitination level. (A and B) m^6^A-deficient virion RNA increased MDA5 binding. MDA5-conjugated magnetic beads were incubated with the RNAs of m^6^A-deficient and wt ALV-J. (A) One aliquot of beads was analyzed using western blotting. (B) RNA bound to magnetic beads was quantified using RT-qPCR. Results were normalized as the ratio between immunoprecipitated RNA from m^6^A-deficient and wt ALV-J. (C) The ubiquitination analysis of MDA5. The m^6^A-deficient ALV-J virion RNA enhanced MDA5 ubiquitination compared with the wt ALV-J RNA. Similarly, 1 μg each of pFlag-MDA5 and pHA-Ub was transfected into DF-1 cells after 24 h, and 10^8^ RNA copies of m^6^A-deficient and wt ALV-J virions were transfected into DF-1 cells. Ubiquitination of MDA5 was analyzed using an anti-Flag blot. *: p < 0.05, **: p < 0.01, ***: p < 0.001, ****: p < 0.0001; ns: no significant difference.

To further explore the mechanisms by which m^6^A inhibits the innate immune response to viral RNA, we directly compared the binding affinity of m^6^A-sufficient (HLJ09SH05) and m^6^A-deficient (SH5-FTO-OE) RNAs with MDA5. Briefly, cells expressing Flag-tagged MDA5 were lysed and pulled down with magnetic beads conjugated with Flag antibodies (Fig 5A). Then complexes were divided equally, and mixed with each of the virion RNAs, and the RNA in the complex was quantified using RT-qPCR. The amount of SH5-FTO-OE RNAs pulled down by MDA5 was approximately thrice that of HLJ09SH05 RNAs pulled down by MDA5 (Fig 5B). This data indicated that m^6^A deficiency enhanced the binding of ALV-J RNA to MDA5. After binding to RNA, MDA5 undergoes significant conformational changes, enabling ubiquitination of MDA5, which is crucial in activating its IFN-I signaling pathway [36]. To test whether m^6^A-deficient ALV-J RNA can promote MDA5 ubiquitination, the pFlag-MDA5 and pHA-Ub plasmids were co-transfected into DF-1 cells for 24 h, and SH5-FTO-OE and HLJ09SH05 RNAs were separately transfected into the cells. Western blotting result showed that SH5-FTO-OE RNA had a stronger ability to promote MDA5 ubiquitination compared to that of HLJ09SH05 RNA (Fig 5C). The above results demonstrate that m^6^A-deficient ALV-J RNA enhances its binding ability with MDA5 and promotes MDA5 ubiquitination.

### ALV-J m^6^A-deficient RNA affects its replication and the IFN-β expression levels *in vitro* and *in vivo*

**Fig 6:**
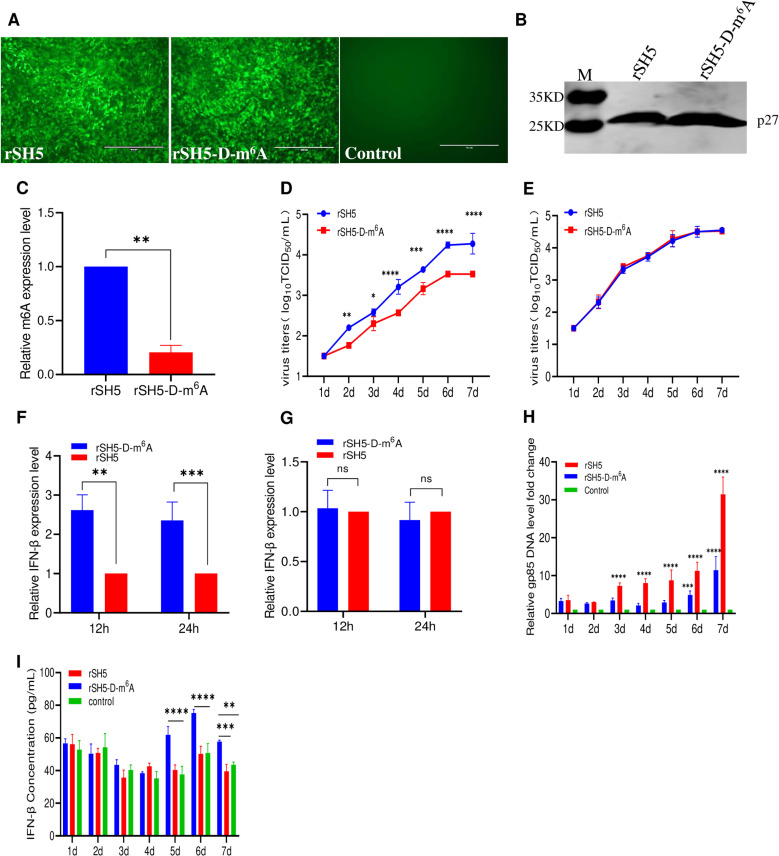
The deficiency of m^6^A in the ALV-J genome attenuated its replication ability. (A) IFA assay. DF-1 cells are infected with wt ALV-J rSH5 and m^6^A-deficient virus rSH5-D-m^6^A for 72 h, and then detected with the 4A3 monoclonal antibody and analyzed using a fluorescence microscope, rSH5 as reference. Scale bar: 400 μm. (B) Western blotting. The wt ALV-J rSH5 and the m^6^A-deficient virus rSH5-D-m^6^A were detected with the 2E5 monoclonal antibody, rSH5 was the reference. (C) Relative m^6^A levels in the RNAs from rSH5-D-m^6^A and rSH5 virions. The m^6^A levels for each virion RNA were quantified using an m^6^A methylation kit. (D) The replication abilities of rSH5-D-m^6^A and rSH5 were detected using TCID_50_ *in vitro*. DF-1 cells were incubated with SH5-D-m^6^A and rSH5 at an MOI of 0.01 and harvested and quantified at 1, 2, 3, 4, 5, 6, and 7 dpi. (E) The replication abilities of rSH5-D-m^6^A and rSH5 were determined using TCID_50_ *in vitro*. MDA5-KO DF-1 cells were incubated with rSH5-D-m^6^A and rSH5 at an MOI of 0.01 and harvested and quantified at 1, 2, 3, 4, 5, 6, and 7 dpi. (F) rSH5-D-m^6^A and rSH5 induced *IFN-β* mRNA expression after infecting DF-1 cells. DF-1 cells were infected with rSH5-D-m^6^A and rSH5. At 12 and 24 hpi, cells were collected to analyze *IFN-β* mRNA levels using RT-qPCR. (G) rSH5-D-m^6^A and rSH5 induced *IFN-β* mRNA levels after infecting MDA5-KO DF-1 cells. MDA5-KO DF-1 cells were infected with rSH5-D-m^6^A and rSH5. At 12 and 24 hpi, cells were collected to analyze *IFN-β* mRNA levels using RT-qPCR. (H and I) 1-day-old SPF chickens were intraperitoneally inoculated with rSH5-D-m^6^A and rSH5 (n = 10, dose = 10^4^ TCID_50_). (H) Viral loads in whole-blood samples were measured using RT-qPCR. (I) The IFN-β levels were also evaluated using a chicken IFN-β ELISA kit at different time points.

Owing to the m^6^A modification of RNA being reversible [37,38], to determine the impact of m^6^A modification in HLJ09SH05 genomic RNA on virus replication ability, we first rescued the wt ALV-J (rSH5) and m^6^A-deficient ALV-J virus (rSH5-D-m^6^A) by mutating all m^6^A modification sites in the *Env* and *3*′*UTR* regions in DF-1 cells. The IFA results indicated that DF-1 cells inoculated with rSH5-D-m^6^A and rSH5 showed specific green fluorescence, uninfected cells did not show such fluorescence (Fig 6A). Western blotting results showed that rSH5-D-m^6^A and rSH5 produced a specific 27-kDa band with the 2E5 monoclonal antibody [39] (Fig 6B). Additionally, RNA was extracted from both viruses for m^6^A modification level analysis. ELISA result showed that the m^6^A modification levels in rSH5-D-m^6^A RNA were approximately 80% lower than those of rSH5 RNA (Fig 6C). These results indicate the successful rescue of the parental virus rSH5 and m^6^A-deficient virus rSH5-D-m^6^A.

Subsequently, to assess the replication capacity of the rSH5 and the rSH5-D-m^6^A, DF-1 and MDA5-KO DF-1 cells were inoculated with rSH5 and rSH5-D-m^6^A, and cell samples were collected from 1 to 7 dpi. The 50% tissue infection dose (TCID_50_) results showed that the titer of the rSH5-D-m^6^A was reduced by approximately 1.8- to 6.4-fold at 2–7 dpi compared with that of the rSH5 in DF-1 cells (Fig 6D). However, in MDA5-KO DF-1 cells, there were no significant differences in the viral titer between both viruses from 1 to 7 dpi (Fig 6E). Furthermore, we examined *IFN-β* expression levels with rSH5 and rSH5-D-m^6^A in DF-1 and MDA5-KO DF-1 cells. The results showed that in DF-1 cells, rSH5-D-m^6^A induced a 2.4–2.6 fold increase in *IFN-β* expression levels compared to that of rSH5 (Fig 6F). In contrast, there were no significant differences in the *IFN-β* expression levels induced by both viruses in MDA5-KO DF-1 cells (Fig 6G). These results indicate that lack of m^6^A modification in the ALV-J genome RNA inhibits viral replication and induces a significantly higher level of *IFN-β* expression in DF-1 cells and further demonstrate that m^6^A modification of ALV-J RNA escapes recognition by MDA5.

To investigate the impact of m^6^A modification in the ALV-J genome on the replication of ALV-J and the induced expression levels of *IFN-β in vivo*, 10^4^ TCID_50_ of rSH5 and rSH5-D-m^6^A were intraperitoneally injected into 1-day-old specific pathogen-free (SPF) chickens, respectively, and blood samples were collected between 1 and 7 dpi. RT-qPCR results showed that the viral loads of the rSH5 group were 2.2–3.8-fold higher than those of the rSH5-D-m^6^A group (Fig 6H). The ELISA result indicated that rSH5-D-m^6^A induced IFN-β expression in SPF chicken similar to that induced by the rSH5 group between 1 and 4 dpi. However, at 5–7 dpi, the IFN-β expression levels in the rSH5-D-m^6^A group were higher than those in the rSH5 and control groups (Fig 6I). These results also show that m^6^A-deficient ALV-J RNA inhibits viral replication and induces significantly higher IFN-β expression *in vivo*.

## Discussion

**Fig 7:**
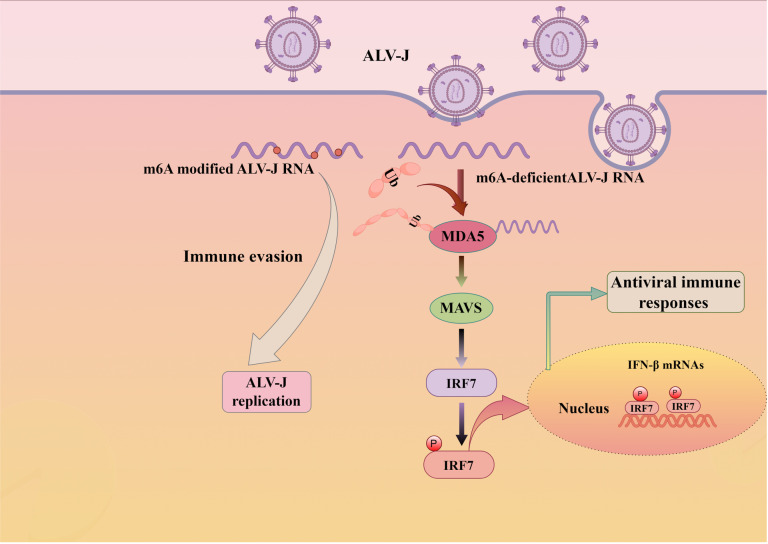
The model of that ALV-J RNA evades innate immune surveillance. ALV-J featuring m^6^A-modified RNA (blue dots) circumvents innate sensing in infected cells, effectively escaping immune detection. Conversely, ALV-J with m^6^A-deficient RNA, upon entering cells, enhances the interaction between MDA5 and viral RNA and MDA5 ubiquitination, then triggers the recruitment of the adaptor protein MAVS, which subsequently leads to IRF-7 phosphorylation ((indicated by the letter P). This process culminates in the formation of IRF-7 homodimers and/or heterodimers, their translocation into the nucleus, and ultimately, the expression of type I IFNs. This picture was drawn by Figdraw.

Recent reports indicate that the m^6^A modification of RNA, as the most common mRNA modification, is significant in the life cycles of numerous viruses and the innate immune response to viral infection [29–31,40]. In this study, we found that ALV-J only induces a weak innate immune response, and its genome is enriched with m^6^A modifications in the *Env* and *3’ UTR* regions. Further analysis revealed that the production of IFN-β is almost undetectable in the presence of m^6^A modifications in the ALV-J genome. More importantly, our result found that the m^6^A modification of ALV-J RNA primarily weakens MDA5 recognition of viral RNAs, diminishes the binding between MDA5 and viral RNA, and reduces MDA5 ubiquitination to inhibit IFN-β expression (Fig 7). These findings preliminarily elucidate the mechanisms by which m^6^A modifications enable ALV-J to evade host immune defenses and establish persistent infections.

Viruses need to evade the host’s innate immune response for infection establishment and persistence. It is reported that HIV infection in cells cannot effectively activate the innate immune response, causing the establishment of persistent infection [41]. Another retrovirus, Simian immunodeficiency virus, is also a poor inducer of innate immunity in pig-tailed macaques, African green monkeys, and mandrills, causing long-term infection [42,43]. In addition, our study proved that ALV-J, as a simple retrovirus, was not effective in inducing the innate immune response after infecting DF-1 cells, chicken primary macrophages, and SPF chickens. These data suggest that retroviruses are poor inducers of innate immune responses, preventing the host from clearing the virus and leading to persistent infection.

Viruses usually evade host immune surveillance through protein and nucleic acid modifications [44–46]. Among these, m^6^A modification of nucleic acids affects mRNA metabolism, translation, and splicing, influencing virus replication, and also plays a critical role in the innate immune response [47,48]. Studies have reported that m^6^A modifications occur in the genomic RNAs of viruses such as HCV, ZIKV, HIV, HSV-1, and HBV [49–55]. Ji et al. recently predicted the m^6^A modification of ALV-J RNA using SRAMP software. However, this result was not further validated [56]. In this study, we systemically confirmed the presence of m^6^A modifications in ALV-J RNA using MeRIP-seq and m^6^A methylase drug inhibition experiments, further identifying its primary localization in the *Env* and *3*′*UTR* regions. Furthermore, we rescued the virus by mutating all m^6^A modification sites in the *Env* and *3*′*UTR* regions, resulting in an 80% decrease in genomic m^6^A modification levels of ALV-J. Additionally, we performed m^6^A sequencing of the genomes of ALV-A and ALV-B, discovering that m^6^A is also mainly concentrated in the *Env* and *3’ UTR* regions (S2 Fig). Notably, previous study has reported that the m^6^A modifications in the genomic RNA of human immunodeficiency virus (HIV) are also enriched in the *Env* and *3’ UTR* regions [57], suggesting that m^6^A modifications in the genomes of retroviruses are focused in these areas. This is similar to the pattern observed in flavivirus genomes, where m^6^A modifications are primarily concentrated in the last viral gene.

The binding of the protein encoded by *Env* to its specific receptor on the target cell is the first and critical step in determining viral infection [2,54,58,59]. *Env* is the most variable gene in ALV-J [7,60,61], however, a comparative analysis of its nucleotide sequences from multiple strains of ALV-J revealed that 92.3% (12 out of 13) of the m^6^A motifs were highly conserved. Further analysis indicates that the relatively conserved *E* element within the *3’UTR* region is another m^6^A enrichment site, which is closely associated with tumorigenicity [62,63]. This suggests that the ALV-J genome m^6^A modification sites are relatively conserved. Studies on human respiratory syncytial virus also revealed that despite the *G* gene having the highest genetic variability, the m^6^A modification sites within it are highly conserved [64]. This suggests that m^6^A modification sites in viral genes may provide an evolutionary advantage for viral infection, replication, and transmission, causing their selective retention.

To explore the potential role of m^6^A modification in ALV-J-induced weak innate immunity, in this study, we obtained m^6^A-deficient viruses through the FTO-OE DF-1 cell line or by mutating the m^6^A modification sites of ALV-J genome. These two viruses and their RNAs induced higher *IFN-β* expression levels in the different cells compared to that of the wt virus and its RNA. These results further demonstrate that m^6^A modification is crucial in inhibiting the *IFN-β* production after ALV-J infects the host. Similar phenomena have also been observed in viruses such as HIV, HCV, and hMPV [65–68]. Recent studies suggest that epitranscriptomic modifications of RNA contribute to viral infection by assisting RNA to escape recognition by PRRs. For example, HIV-1 RNA uses 2’-O-methylation to escape MDA5 recognition, inducing lower IFN-I levels [69], m^6^A modifications assist VSV, RSV, hMPV, and HIV RNAs in evading RIG-I recognition, escaping the innate immune response [67,68,70]. However, chickens lack RIG-I, prompting the investigation of how the m^6^A modification of ALV-J genomic RNA aids RNA in evading PRRs recognition. Our results confirmed that m^6^A modification can weaken the recognition and binding of MDA5 to ALV-J genomic RNA and its ubiquitination. These data emphasize that chicken MDA5 can compensate for the function of RIG-I and also suggest that m^6^A modification in RNA is a molecular marker of host innate immunity, which can be used by RLR pattern recognition receptors to distinguish between self and non-self RNAs.

m^6^A modifications not only promote viral replication by evading innate immunity but also affect mRNA metabolism, translation, and splicing. For example, the m^6^A modifications on the mRNA of the highly mutated G protein of the RSV enhance the stability of the mRNA, promoting its translation into more G protein. This leads to increased incorporation of G protein into viral particles, resulting in the production of more infectious viral particles [64]. Additionally, the increased soluble G protein can influence leukocyte migration, thereby regulating the host immune response to RSV [71]. In this study, during the early stage of infection, there is no difference in IFN-β induction between rSH5 and rSH5-D-m^6^A, but the level of gp85 mRNA is higher in rSH5. It is speculated that the m^6^A modification in the mRNA of the Env gene may enhance its stability, promote the translation of Env protein and viral assembly, and increase the production of infectious viral particles. Moreover, studies show intracellular Env inhibits IFN-β production, exerting immunosuppression [72], which could explain the reduced IFN-β at 7 dpi, but further verification is needed.

In conclusion, our study demonstrated the pivotal role of m^6^A modifications within the *Env* and *3’UTR* regions of ALV-J genomic RNA in dampening the host’s innate immune defenses. Specifically, these modifications curtail the capacity of MDA5 to identify viral RNA, thereby hindering a robust immune reaction. Our findings not only elucidated the molecular mechanism by which m^6^A modification of ALV-J genomic RNA caused the virus evasion of innate immune responses but also provided insights into further elucidating the infection and pathogenic mechanisms of ALV.

## Materials and methods

### Ethics statement

All animal experiments were approved by the Committee on the Ethics of Animal Experiments of Harbin Veterinary Research Institute (HVRI), Chinese Academy of Agricultural Sciences (CAAS) (240130–01-GR). Specific pathogen-free (SPF) chickens were purchased from the Experimental Animal Centre of the HVRI and housed in negative-pressure isolators with adequate food and light. All animal procedures were performed according to the international standards for animal welfare.

### Cells, viruses, and plasmids

HEK293T and DF-1 cells were cultured in Dulbecco’s modified Eagle’s medium (DMEM) (L110KJ, BasalMedia, China) supplemented with 10% (vol/vol) fetal bovine serum (FBS) (FDN500, Excell Bio, China) in a humidified incubator with 5% CO_2_ at 37 °C. DF-1 cells were cultured in DMEM containing 10% FBS. Chicken primary macrophages were prepared from the bone marrow of 3-week-old SPF chickens according to a previously described method [73] and cultured in an RPMI-1640 medium (C11875500BT, Gibco, USA) supplemented with 5% FBS, 5% chicken serum (S9080-500ML, Solarbio, China), 10% tryptose phosphate broth (T8782-500G, Sigma, USA), 1% sodium pyruvate (11360–070, Gibco, USA), and 0.1% β-mercaptoethanol (21985023, Gibco, USA). DF-1 and chicken primary macrophages cells were cultured in a humidified incubator with 5% CO_2_ at 38.5 °C. The epidemic strain HLJ09SH05 was identified and maintained at the Harbin Veterinary Research Institute, CAAS (Harbin, China).

The pLVX-IRES-Puro, psPAX2, pMD2.G, CRISP/Cas9, pHA-Ub, pFlag-MDA5, pCAGGS, and pBluescript II KS(+) plasmids were maintained in our laboratory. FTO (GenBank accession number: HM050377.1) was amplified from the cDNA of DF-1 cells through RT-PCR and was inserted into the pLVX-IRES-Puro plasmid with a Flag tag at the C-terminus to construct the FTO-OE DF-1 cell line. This plasmid was named pLVX-FTO-Flag.

To construct a pBlueSH5 infectious clone, the full-length cDNA of HLJ09SH05 was inserted into the pBluescript II KS(+) plasmid as described in the previous study [39,62]. Based on MeRIP-seq data, 16 potential m^6^A sites were found in the *Env* and *3’UTR* regions of the ALV-J genome, which were mutated using synonymous mutations. The resultant plasmid was named pBlueSH5-D-m^6^A. All plasmid sequences were confirmed through DNA sequencing. The primer sequences for all oligonucleotides used in this study are available upon request.

### MeRIP-seq

First, the total RNAs from DF-1 cells infected with HLJ09SH05 were extracted, and the mRNA with poly-A tails within the total RNAs were enriched using Oligo-dT beads. Subsequently, the enriched mRNAs were randomly broken into approximately 100 bp segments by adding divalent cations. The randomly fragmented RNAs were divided into two parts: one part was used to construct a traditional transcriptome sequencing library as a control, while the other part was added to magnetic beads coated with m^6^A antibodies to enrich for m^6^A-modified mRNA fragments. Using the enriched mRNA as a template, we synthesized cDNA with random primers and ligated it using splint adapters. Then, we performed PCR amplification on the adapter-ligated products to construct the m^6^A-seq library. After constructing the library, we used agarose gel electrophoresis to detect RNA contamination and degradation and simultaneously employed Ubit 3.0 to accurately measure the RNA concentration. Once both sequencing libraries were successfully constructed and passed quality control, we subjected them to high-throughput sequencing. The raw sequencing data obtained were then filtered to produce high-quality sequencing data (clean data). Subsequently, we aligned the clean data to the reference genome of the project species to obtain comprehensive transcriptome information, and performed gene expression quantification as well as GO and KEGG Pathway analyses. Additionally, we conducted whole-genome de novo peak calling on the alignment results to investigate the binding preferences of proteins on RNA. Finally, we performed motif analysis on these binding sites to further reveal their functions and characteristics.

### Generation of m^6^A-deficient ALV-J from DF-1 cells treated with DAA

DF-1 cells were pre-treated with 10 μM DAA or DMSO for 4 h before inoculation with 0.01 MOI HLJ09SH05. Subsequently, the cells were supplemented with DMEM containing 10 μM DAA and 2% FBS and subjected to continuous culturing for 4 days. The cell supernatants were collected for virus purification, and the RNA m^6^A modification was analyzed using an m^6^A RNA methylation assay kit.

### Colorimetric quantification of viral m^6^A methylation

According to the manufacturer’s instructions, the m^6^A modification levels of virion RNA were quantified using an m^6^A RNA methylation assay kit (ab185912, Abcam, UK). Briefly, the same amount of sample RNA, negative control, and positive control were separately added into the wells and incubated at 37 °C for 90 min, followed by three washes with 1× wash buffer. Subsequently, 50 µL of diluted capture antibody (1:1000) was added to each well and incubated at room temperature for 60 min, followed by three washes with 150 µL of 1× wash buffer. Next, 50 µL of diluted detection antibody (1:2000) was added to each well, followed by incubation at room temperature for 30 min, and each well was washed four times with 150 µL of 1× wash buffer. Furthermore, 50 µL of diluted enhancer solution (1:3000) was added to each well and incubated at room temperature for 30 min, followed by five washes with 150 µL of 1× wash buffer. Similarly, 100 µL of substrate solution was added to each well, followed by incubation at room temperature in the dark for 5 min, and 100 µL of stop solution was added to each well to terminate the enzyme reaction. Finally, the absorbance was measured and recorded at a wavelength of 450 nm.

### Generation of FTO-OE DF-1 cell line

To obtain a DF-1 cell line stably expressing FTO, we first co-transfected the recombinant plasmid pLVX-FTO-Flag along with the helper plasmids psPAX2 and pMD2.G into HEK293T cells. 48 hours post-transfection (hpt), we collected the cell supernatants and centrifuged them at 5,000×g for 10 min at 4°C. The centrifuged supernatants were then filtered through a 0.45-μM filter and subsequently inoculated into DF-1 cells. After 96 h, the inoculated cells underwent three consecutive rounds of selection using 1.5 μg/mL puromycin to select stably expressing cells. Once the stably expressing cell line was established, we utilized western blotting and IFA to detect the expression of FTO.

### Virus production and purification

The wt and FTO-OE DF-1 cells were infected with ALV-J at an MOI of 0.01, and the cell culture supernatants were harvested. First, the cell culture supernatants were clarified through centrifugation at 1,500 × *g* for 5 min to remove cellular debris, and this supernatant virus was passed through a 0.45-μm syringe filter. Furthermore, the virus was concentrated through a 25% (wt/vol) sucrose cushion via centrifugation at 28,000 × *g* for 2 h at 4 °C in a type SW32Ti rotor (Beckman, Brea, CA). Finally, the pellet was resuspended in DMEM.

### RNA transfection of cells

For RNA transfection, 5×10^5^ DF-1 cells, MDA5-KO DF-1celLs, and chicken primary macrophage cells were then transfected with 10^8^ copies ALV-J RNA using TransIT mRNA transfection kits (MIR 2225, Mirus, USA) according to the manufacturer protocol. At 12 hpt, cells were harvested for measuring *IFN-β* and *GAPDH* mRNA by RT-qPCR.

### RT-PCR

According to the manufacturer’s instructions, RNA was extracted from cells and virion RNA from purified virus using the RNAiso Plus reagent (9108, TaKaRa, Japan). The RNA was reverse-transcribed into cDNA using a Reverse Transcription Kit (R223-01, Vazyme, China). The quantity of each cDNA was determined through RT-qPCR using the THUNDERBIRD SYBR qPCR Mix Kit (QPS-201, TOYOBO, Osaka, Japan) and analyzed with the QuantStudio 5 system (Applied Biosystems, USA). The specific primers for *IFN-β*, *ZAP, Env, ISG12–1, Mx-1*, and *IFITM3* were designed according to references [74] and synthesized by Invitrogen (Shanghai, China). The relative mRNA levels of these genes were normalized to chicken *GAPDH* mRNA levels in each sample.

### RNA interference

Three siRNAs specifically targeting chicken TLR7 (siTLR7–1: 5′- GGUGAUGACAGAAUUGGUUdTdT-3′; siTLR7–2: 5′-CCAGAACUCAAGAUACUAAdTdT-3′; siTLR7–3: 5′-CCACCCAACUUAUCUUCAAdTdT-3′), chicken MDA5 (siMDA5–1: 5′- GGUAUCAAGUUAUUGGCUUdTdT-3′; siMDA5–2: 5′-GCAGAACACUUGAAGAAAUdTdT-3′; siMDA5–3: 5′-CCGCCAGAAGAGUAUUUAAdTdT-3′) and a scramble negative control siRNA (5′-UUCUCCGAACGUGUCACGUdTdT-3′) were synthesized by Seven Biotech (Beijing, China). According to the manufacturer’s instructions, the siRNA transfections were performed in DF-1 cells using X-tremeGENE siRNA Transfection Reagent (04476115001, Roche, USA). After 24 h of transfection, cells were harvested to determine the knockdown efficiency using RT-qPCR. In addition, after 24 h of siRNA transfection, DF-1 cells were transfected with viral RNA or inoculated with ALV-J for subsequent analyses.

### Generation of MDA5-KO DF-1 cell line

CRISPR/Cas9 was used to knock out the MDA5 gene in DF-1 cells. The sgRNA (5′-CGGATGGTTCACTGAATTCC-3′) was designed using E-CRISPR (http://www.e-crisp.org/E-CRISP/designcrispr.html). The DNA fragments containing the U6 promoter, target gRNA, and the gRNA scaffold were fused using overlap PCR and inserted into the pMD-18T vector (6011, TaKaRa, Japan). Subsequently, the gRNA-carrying pMD-18T and pMJ920 plasmids were transfected into DF-1 cells using the TransIT-X2 delivery system (MIR 6000, Mirus, USA) according to the manufacturer’s instructions. Cells with green fluorescence were sorted into a 96-well plate using flow cytometry, and monoclonal cells were identified through sequence analysis, RT-qPCR, and poly (I:C) stimulation.

### CCK8 assay

To detect MDA5-KO DF1 cell proliferation rates using the CCK8 kit, the detection principle of this kit is that WST-8 in the CCK8 reagent, with the assistance of the electron carrier (1-Methoxy PMS), can be reduced by dehydrogenases within cells to produce a highly water-soluble yellow formazan dye, which is directly proportional to the number of viable cells. The simplified procedure is as follows: First, 100μL of DF-1 cell and MDA5-KO DF1 cell suspensions are separately inoculated into a 96-well plate and incubated for 24 h in a 38.5°C culture incubator. Then, 10μL of CCK8 solution is added to each well and incubated for another 2 hours. Finally, the absorbance value of each well at a wavelength of 450 nm were measured by using a microplate reader, and the cell proliferation rate based on the absorbance values.

### Determination of IFN-β using ELISA

The IFN-β levels in the serum of ALV-J- or mock-infected chicken were determined using a chicken IFN-β ELISA kit (SEA222Ga, Cloud-Clone, China), following the manufacturer’s instructions.

### Protein-RNA pull down

After transfecting pFlag-MDA5 into HEK293T cells for 48 h, the cells were washed thrice with PBST and lysed with NP40 buffer containing 1 mM phenylmethanesulfonyl fluoride protease inhibitors on ice for 30 min; the cell lysate was centrifuged at 12,000 × *g* for 10 min at 4 °C. Subsequently, 80 μL of the cell lysate was taken and mixed with 20 μL of 5×SDS loading buffer, followed by denaturation at 100 °C for 10 min as input control. The remaining cell lysate was incubated with 20 μL of Anti-FLAG(R) M2 beads (M8823-5ML, Sigma-Aldrich, USA) for 1 h at room temperature on a 4D rotator. The cell lysate bound to the Anti-FLAG(R) M2 magnetic beads was divided into four 1.5 mL EP tubes, with two tubes receiving 10^8^ copies of HLJ09SH05 RNA and SH5-FTO-OE RNA, followed by incubation for 1 h at room temperature, while the other two tubes served as negative controls. The samples were washed five times with 0.5 mL of NP40 lysis buffer, each time for 10 min, and the supernatants were discarded. One negative control was mixed with 80 μL of PBS and 20 μL of 5×SDS loading buffer and denatured at 100 °C for 10 min. The other three samples were mixed with 200 μL of RNAiso plus for RNA extraction and reverse transcription, followed by RT-qPCR to detect RNA levels.

### Ubiquitination assay

To investigate whether m^6^A-deficient ALV-J RNA can increase the ubiquitination modification of MDA5, 1 μg of pFlag-MDA5 and pHA-Ub or pCAGGS plasmids were transfected into DF-1 cells for 24 h, followed by the transfection of 10^8^ RNA copies. After 12 h of RNA transfection, the cells were washed thrice with PBS and lysed with 500 μL of NP40 lysis buffer on ice for 30 min. The cell lysate was centrifuged at 12,000 × *g* for 10 min at 4 °C, and 80 μL of the cell lysate was mixed with 20 μL of 5×SDS loading buffer and denatured at 100 °C for 10 min as an input control. The remaining sample was incubated with 20 μL of Anti-FLAG(R) M2 magnetic bead on a 4D rotator for 8 h at 4 °C. After washing the cells thrice with precooled PBST, 80 μL of PBS and 20 μL of 5×SDS loading buffer were mixed and denatured at 100 °C for 10 min. The samples were detected and analyzed using western blotting.

### Rescue of recombinant viruses

The plasmids pBlueSH5 and pBlueSH5-D-m^6^A were transfected into DF-1 cells using the TransIT-X2 Dynamic Delivery System (MIR6000, Mirus Bio LLC, USA). After 7 days, the culture supernatant containing the viral stock was harvested and blindly passed to the next generation of DF-1 cells. The rescued viruses were named rSH5 and rSH5-D-m^6^A, respectively. Subsequently, the titers of the rescued recombinant viruses were determined using the Reed and Muench method [75].

### IFA

First, after inoculating DF-1 cells with 200 TCID_50_ of rSH5 and rSH5-D-m^6^A for 72 h, the cells were fixed with cold solute ethanol at room temperature for 15 min. Subsequently, the cells were washed thrice with PBST and incubated with 4A3 (mouse anti-gp85 antibody, diluted at 1:200 ratio) at 37 °C for 1 h [39]. Next, after washing the cells thrice with PBST, they were incubated with a 1:200 dilution of fluorescein isothiocyanate-conjugated secondary antibody (FITC-conjugated goat anti-mouse IgG) at 37 °C for 1 h. Finally, DF-1 cells were observed using a fluorescence microscope (TU-80, Nikon, Tokyo, Japan). Normal DF-1 cells were used as a negative control.

### Experimental infections

To detect the innate immune response induced by the HLJ09SH05 infection in SPF chickens, 20 1-day-old SPF chickens were divided into two groups, with 10 chickens in each group, and were maintained in different negative pressure isolators. One group was infected with 10^4^ TCID_50_ of HLJ09SH05 via intraperitoneal injection; the other group received intraperitoneal PBS injection. Blood samples were collected on 1, 2, 3, 4, 5, 6, and 7 dpi to assess viremia and IFN-β levels using RT-qPCR and ELISA, respectively.

To investigate whether the m^6^A modification of the ALV-J genome affects the replication ability of the ALV-J and the induction of innate immunity, 10^4^ TCID_50_ of rSH5 and rSH5-D-m^6^A were injected intraperitoneally into 1-day-old SPF chickens. Blood samples were collected between 1 and 7 dpi to assess viremia and IFN-β levels.

### Statistical analysis

GraphPad Prism software (version 7.03, GraphPad Software, San Diego, CA, USA) was used for the statistical analysis. The two-way ANOVA was used to assess differences between groups. Statistical significance was established at *P* < 0.05. * *P <* 0.05; ** *P <* 0.01; *** *P <* 0.001; **** *P <* 0.0001.

## Supporting information

S1 Fig TLR7 is not the primary RNA sensor for recognizing m^6^A-defective ALV-J RNAs. (A) Validation of the optimal siRNA targeting TLR7 using RT-qPCR. (B) The impact of TLR7 gene knockdown on the expression of *IFN-β* mRNA in chicken primary macrophages induced by m^6^A-defective viruses. Chicken primary macrophages were transfected with 2 μg of siTLR7–1 or a negative siRNA control (siSc). Subsequently, the cells were infected with m^6^A-defective or wt ALV-J for 24 h. (C) The effect of TLR7 knockdown on the levels of *IFN-β* mRNA in chicken primary macrophages induced by m^6^A-defective viruses. Chicken primary macrophages were transfected with 2 μg of siTLR7–1 or a negative siRNA control (siSc), followed by transfection with 10^8^ copies of m^6^A-defective or wt ALV-J for 12 h.
(TIF)

S2 Fig Distribution of m^6^A peaks in the ALV-A and ALV-B genome. The total RNA of DF-1 cells infected with RAV-1 and RAV-2 was separately extracted at 4 dpi and subjected to m^6^A-specific antibody immunoprecipitation, followed by high-throughput sequencing (MeRIP-seq). Purple areas illustrate the distribution of m^6^A immunoprecipitation reads aligned to the ALV-A mRNAs, while the baseline signal from input samples is depicted as a continuous line. Yellow areas demonstrate the distribution of m^6^A immunoprecipitation reads aligned to the ALV-B mRNAs, while the baseline signal from input samples is depicted as a continuous line.
(TIF)

S1 Data Microsoft Excel workbook provided source data matrices and associated statistical computations used to generate the graphical representations in Figures. (XLSX)
