# Supplementary figures and images for: N6-methyladenosine modification of the subgroup J avian leukosis viral RNAs attenuates host innate immunity via MDA5 signaling
Source: PLoS Pathog. 2025 Apr 8;21(4):e1013064. doi: 10.1371/journal.ppat.1013064 (PMC12043233; doi:10.1371/journal.ppat.1013064)

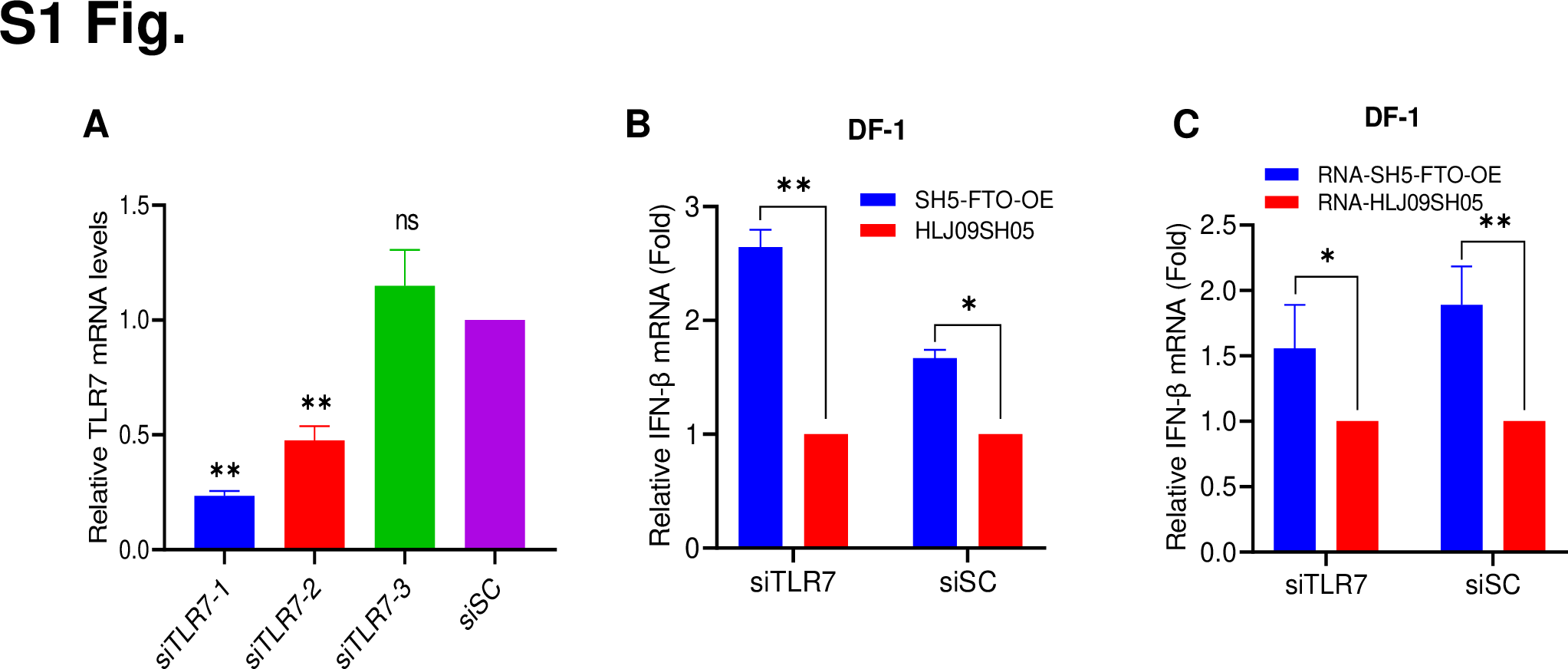

Supplement: S1 Fig — (A) Validation of the optimal siRNA targeting TLR7 using RT-qPCR. (B) The impact of TLR7 gene knockdown on the expression of IFN-β mRNA in chicken primary macrophages induced by m6A-defective viruses. Chicken primary macrophages were transfected with 2 μg of siTLR7–1 or a negative siRNA control (siSc). Subsequently, the cells were infected with m6A-defective or wt ALV-J for 24 h. (C) The effect of TLR7 knockdown on the levels of IFN-β mRNA in chicken primary macrophages induced by m6A-defective viruses. Chicken primary macrophages were transfected with 2 μg of siTLR7–1 or a negative siRNA control (siSc), followed by transfection with 108 copies of m6A-defective or wt ALV-J for 12 h. (TIF) [file ppat.1013064.s001.tif]

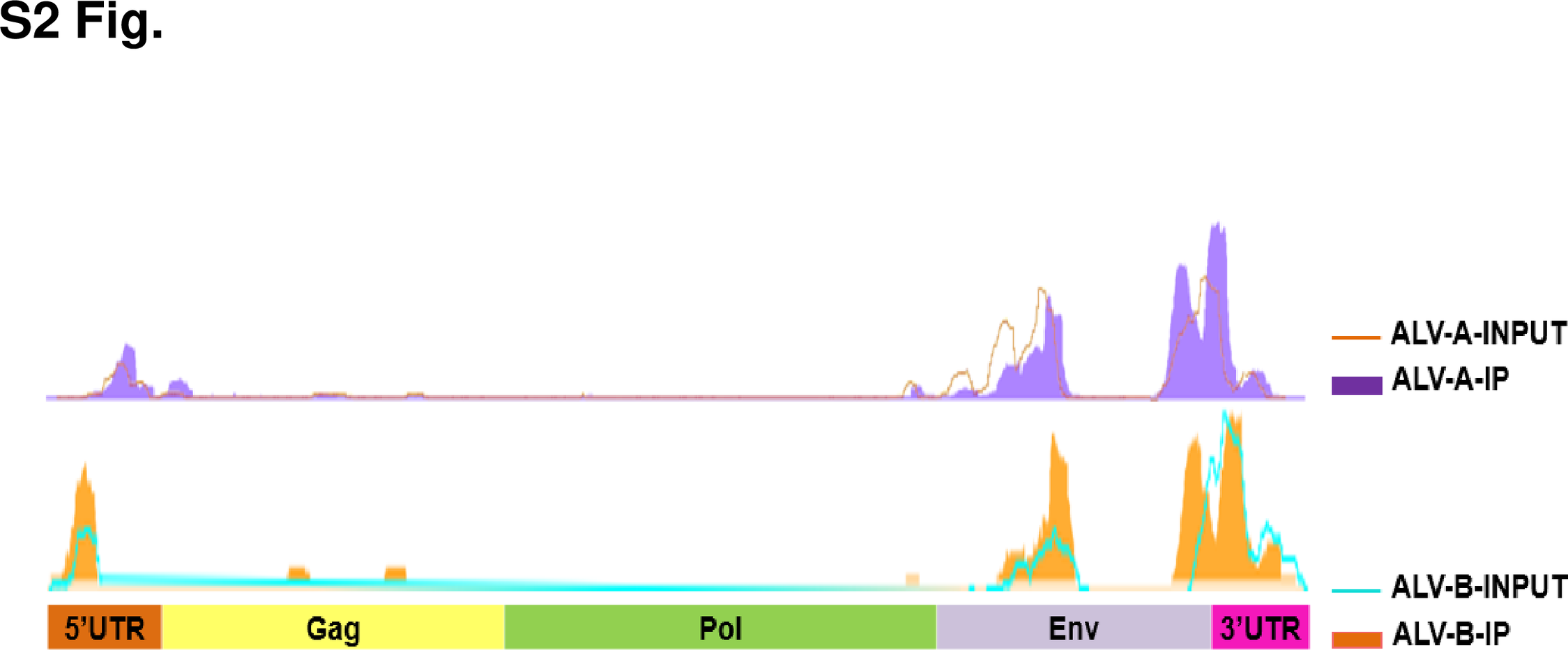

Supplement: S2 Fig — The total RNA of DF-1 cells infected with RAV-1 and RAV-2 was separately extracted at 4 dpi and subjected to m6A-specific antibody immunoprecipitation, followed by high-throughput sequencing (MeRIP-seq). Purple areas illustrate the distribution of m6A immunoprecipitation reads aligned to the ALV-A mRNAs, while the baseline signal from input samples is depicted as a continuous line. Yellow areas demonstrate the distribution of m6A immunoprecipitation reads aligned to the ALV-B mRNAs, while the baseline signal from input samples is depicted as a continuous line. (TIF) [file ppat.1013064.s002.tif]
